# A biomarker risk score predicts adverse cardiovascular outcomes in patients with angina and non-obstructive coronary artery disease

**DOI:** 10.1016/j.ajpc.2026.101701

**Published:** 2026-06-10

**Authors:** Jingwen Huang, Ana Leon, Julia Ossi, Yi-An Ko, Taha Ahmed, Jose R. Medina-Inojosa, Kristen Harris, Ayman Alkhoder, Huiying Yang, Liana Alcaro, Mahmoud Al Kasem, Rafia Lodhi, Ahmed Eldaidamouni, Wesam Hritani, Nisreen Haroun, Muhammet Hasan, Laurence S Sperling, Arshed A. Quyyumi, Puja K. Mehta

**Affiliations:** a Emory Clinical Cardiovascular Research Institute, Division of Cardiology, Department of Medicine, Emory University School of Medicine, Atlanta, Georgia; b Department of Surgery, Medstar Georgetown University, Washington, D.C, USA; c Department of Medicine, Emory University School of Medicine, Atlanta, Georgia; d Department of Biostatistics and Bioinformatics, Rollins School of Public Health, Emory University, Atlanta, Georgia; e William Carey University College of Osteopathic Medicine, Hattiesburg, Mississippi, USA

**Abbreviations and acronyms**
ANOCA: angina with non-obstructive coronary arteries
CAD: coronary artery disease
MACE: major adverse cardiovascular events
CV death: cardiovascular death
NT-proBNP: N-terminal pro-B-type natriuretic peptide
BRS: biomarker risk score
sHR: subdistribution hazard ratio
NRI: net reclassification improvement
IDI: integrated discrimination improvement

## 1 Background

Angina with non-obstructive coronary arteries (ANOCA) is increasingly recognized as a clinically important syndrome rather than a benign condition. Patients with ANOCA are at intermediate risk for major adverse cardiovascular events (MACE) despite the absence of flow-limiting epicardial stenosis and have persistent symptoms and impaired quality of life, comparable to those with obstructive coronary artery disease (CAD) [1,2]. However, risk stratification remains limited in everyday practice as routine clinical variables incompletely identify who among the ANOCA patients are at greatest risk. Recent evidence suggests that biomarkers reflecting distinct pathophysiologic processes may enhance risk stratification in ANOCA and may provide incremental prognostic information because ANOCA likely reflects multiple overlapping biological processes. This study aims to evaluate the individual and combined prognostic value of N-terminal pro-B-type natriuretic peptide (NT-proBNP), galectin-3, and homocysteine in predicting incident MACE and cardiovascular death in patients with ANOCA, hypothesizing that activation of these pathophysiologic pathways will identify high-risk subgroups amenable to more personalized and targeted management.

## 2 Methods

Between May 2004 and August 2014, patients undergoing clinically indicated coronary angiography were enrolled in the Emory Cardiovascular Biobank [3]. For this analysis, we included 559 patients with ANOCA, defined as angina or angina-equivalent symptoms with <50 % epicardial coronary stenosis and no prior exclusionary cardiac conditions. Patients who had obstructive CAD, prior coronary revascularization, heart failure or left ventricular ejection fraction <50 %, cardiomyopathy, significant valvular heart disease, pulmonary hypertension, chronic kidney disease with estimated glomerular filtration rate < 45 mL/min per 1.73 m^2^, cardiac transplantation, cardiac surgery, congenital heart disease, or malignancy were excluded. Demographic and clinical data, collected via questionnaires and medical records, included age, sex, race, body mass index (BMI), ever smoking, diabetes mellitus, hypertension, dyslipidemia (defined as total cholesterol ≥200 mg/dL, low-density lipoprotein >130 mg/dL, high-density lipoprotein <40 mg/dL, or lipid-lowering therapy), statin use, and ever smoked [3]. Plasma NT-proBNP, galectin-3, and homocysteine were measured from fasting blood samples collected at the time of catheterization. The primary outcomes were 5-year MACE, defined as cardiovascular death, myocardial infarction, stroke, heart failure hospitalization, or revascularization, and cardiovascular death. The study was approved by the Emory University Institutional Review Board.

Group differences were evaluated using ANOVA for continuous variables and chi-squared tests for categorical variables. Non-normally distributed biomarkers (NT-proBNP, galectin-3, homocysteine) were compared using the Kruskal-Wallis test in unadjusted analyses and log-transformed for multivariable analyses. Fine-Gray competing risk regression was used to account for non-cardiovascular death, adjusting for age, sex, hypertension, diabetes, dyslipidemia, statin use, and smoking history. To address missing data, k-nearest neighbors (kNN) imputation (k = 5) was performed based on similarity across data points. Model performance was evaluated by C-statistic, net reclassification improvement (NRI), and integrated discrimination improvement (IDI) [4].

## 3 Results

The cohort had a mean age of 59.1±11.9 years, and 49.6 % were female. Median NT-proBNP, galectin-3, and homocysteine levels were 97.5 pg/mL, 13.55 ng/mL, and 9.25 μmol/L, respectively. During median follow-up of 5 years, 77 patients (13.8 %) experienced MACE and 45 (8.1 %) died of cardiovascular causes (Fig. 1A). NT-proBNP was weakly correlated with galectin-3 (r = 0.23, p = 0.012). No statistically significant correlations were found between NT-proBNP and homocysteine, and between Galectin-3 and homocysteine. To capture cumulative biomarker burden, we created a BRS by assigning 1 point for each biomarker above the cohort median, yielding scores from 0 to 3. A BRS of 0 was present in 17.6 % of patients, 1 in 32.6 %, 2 in 30.8 %, and 3 in 19.0 %.

**Fig. 1:**
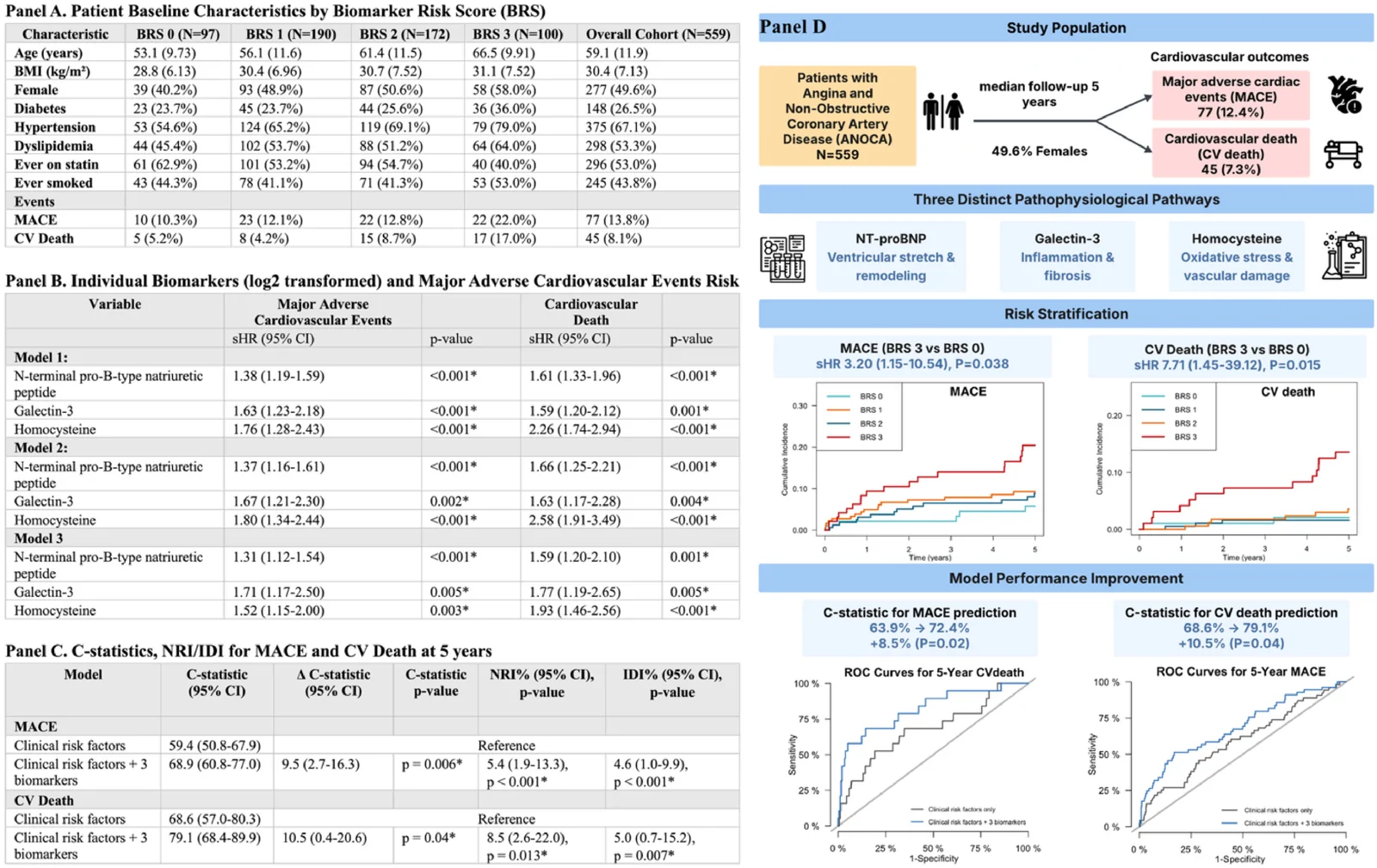
Panel A. BRS was calculated by summing binary scores for each biomarker (NT-proBNP, galectin-3, homocysteine) based on whether levels were above (score = 1) or below the median (score = 0). Values are presented as mean (SD), or N (%). BRS, biomarker risk score; SD, standard deviation; N, number; BMI, body mass index; MACE, major adverse cardiovascular events; CV, cardiovascular; NT-proBNP, N-terminal pro-B-type natriuretic peptide. Panel B. Model 1: Univariable Fine-Gray regression (each biomarker analyzed separately)**.** Model 2: Multivariable Fine-Gray regression (each biomarker analyzed separately, adjusted for age, sex, hypertension, diabetes, dyslipidemia, statin use, and ever smoked). Model 3: Multivariable Fine-Gray regression (all biomarkers entered simultaneously, adjusted for risk factors as above). Abbreviations: sHR, subdistribution hazard ratios; CI, confidence interval. Asterisks indicate statistical significance (*p* < 0.05). sHR represents the difference in hazards per doubling of biomarker measurement. Panel C. Comparison of predictive performance for MACE and CV death between the baseline model (clinical risk factors: age, sex, hypertension, diabetes, dyslipidemia, statin use, and ever smoked) and the enhanced model (clinical risk factors plus NT-proBNP, galectin-3, and homocysteine). Delta (Δ) C-statistic represents the absolute improvement in discrimination when biomarkers are added to the baseline model. CI, confidence interval; NRI, net reclassification improvement; IDI, integrated discrimination improvement; NT-proBNP, N-terminal pro-B-type natriuretic peptide; MACE, major adverse cardiovascular events; CV death, cardiovascular death. Asterisks indicate statistical significance (*p* < 0.05). Panel D. Three biomarkers reflecting distinct pathophysiological pathways (NT-proBNP, galectin-3, homocysteine) were combined into a Biomarker Risk Score (BRS 0-3) with increased MACE and CV death risks observed with higher BRS. Addition of biomarkers to clinical risk factors significantly improved model discrimination for both outcomes. sHR = subdistribution hazard ratio; MACE = major adverse cardiovascular events; CV = cardiovascular; ROC, Receiver operating characteristic curves.

Each biomarker was independently associated with increased risk of MACE and CV death (Fig. 1B). In multivariable Fine-Gray models the cumulative incidence of MACE progressively increased with an increase in BRS, where those with a BRS of 3 had the highest cumulative incidence at 20.8 % at 5 year follow-up, and a sHR of 3.49 (95 % CI: 1.15-10.54, p = 0.027), compared to those with a BRS 0 had a MACE cumulative incidence of 5.8 %. Similarly, for CV death, those with a BRS of 3 exhibited the highest cumulative incidence of 13.0 %, with an adjusted sHR of 7.53 (95 % CI: 1.45-39.12, p = 0.016) compared to patients with a BRS of 0 who had a CV death cumulative incidence of 2.1 % (Fig. 1D). These associations remained significant after mutual adjustment for all three biomarkers and clinical covariates including age, sex, hypertension, diabetes, dyslipidemia, statin use, and ever smoked, indicating that each biomarker contributed independent prognostic information.

Adding NT-proBNP, galectin-3, and homocysteine to clinical risk factors improved prognostic discrimination. For MACE, the C-statistic increased from 59.4 % with clinical variables alone to 68.9 % with biomarkers added. This improvement was accompanied by significant gains in NRI and IDI, indicating that the biomarker-enhanced model improved classification of future risk beyond conventional factors. For cardiovascular death, the C-statistic increased from 68.6 % to 79.1 %, again with significant improvement in reclassification and discrimination. (Fig. 1C and D).

## 4 Discussion

Novel findings of this study are that among patients with ANOCA, elevated levels of NT-proBNP (ventricular stretch and remodeling), galectin-3 (pro-inflammation and fibrosis) and homocysteine (oxidative stress), are independent predictors of adverse CV events, even after adjusting for traditional risk factors. Our study is clinically relevant as all three biomarkers are readily available and prognostic, making the BRS feasible to incorporate into risk assessment of ANOCA patients; however, prospective trials are needed before BRS-guided management can be recommended. Several observations may explain these findings. NT-proBNP reflects myocardial stretch and hemodynamic burden and may identify occult diastolic dysfunction or heart failure with preserved ejection fraction physiology, both of which are plausible contributors to symptoms and prognosis in ANOCA [5]. Galectin-3 is involved in inflammation and fibrosis and may signal adverse vascular remodeling, microvascular dysfunction, or diffuse myocardial remodeling [6]. Homocysteine promotes oxidative stress and endothelial dysfunction, mechanisms that are highly relevant to coronary microvascular disease and abnormal vasomotor regulation [7]. Prior studies have linked ANOCA or microvascular angina with adverse outcomes and with individual biomarkers, but our study extends this work by showing that three markers spanning distinct pathways provide additive prognostic value in the same cohort.

This study includes a large, well-characterized cohort of patients with ANOCA who have angiography-confirmed non-obstructive CAD and rigorous exclusion of conditions that could independently elevate biomarker levels, such as heart failure, chronic kidney disease, or prior revascularization. However, limitations include the absence of coronary function testing to confirm microvascular dysfunction and other vascular endotypes, which could provide further mechanistic insights. Invasive coronary function testing to define microvascular endotypes based on coronary flow reserve, index of microcirculatory resistance, or vasospasm, was performed, precluding subgroup specific associations between biomarkers and outcomes. Median-derived BRS cutoffs are cohort-specific and exploratory, requiring validation against established clinical or data-driven thresholds in independent cohorts. Biomarker levels were measured at a single time point, potentially missing temporal variability. The single-center design may limit generalizability, and future studies should validate these findings in diverse populations and explore longitudinal biomarker trends and the impact of targeted interventions.

## 5 Conclusions

In patients with ANOCA, NT-proBNP, galectin-3, and homocysteine each independently predict adverse cardiovascular outcomes, and the addition of these biomarkers in a biomarker risk score improves prediction of MACE and cardiovascular death. These findings support biomarker-informed risk stratification in ANOCA and provide a rationale for external validation and future biomarker-guided studies. These findings are hypothesis-generating and require prospective external validation before clinical adoption.

## Fundings

Jingwen Huang, MD is supported by Katz preventive cardiology fellowship. Arshed A. Quyyumi, MD is supported by 10.13039/100000002National Institutes of Health through the following grants: 1P01HL154996-04, 5R01HL157311-04, 1R01HL166004-03, R01 AG089759-01, 3P30DK111024-07S2, 5T32 HL130025. Puja K. Mehta, MD is supported by 10.13039/100000002National Institutes of Health: grant NIH R01HL157311.

## CRediT authorship contribution statement

**Jingwen Huang:** Writing – review & editing, Writing – original draft, Visualization, Validation, Methodology, Formal analysis, Conceptualization. **Ana Leon:** Data curation. **Julia Ossi:** Writing – review & editing, Writing – original draft. **Yi-An Ko:** Data curation. **Taha Ahmed:** Writing – review & editing. **Jose R. Medina-Inojosa:** Writing – review & editing. **Kristen Harris:** Writing – review & editing. **Ayman Alkhoder:** Writing – review & editing, Data curation. **Huiying Yang:** Data curation. **Liana Alcaro:** Writing – review & editing. **Mahmoud Al Kasem:** Writing – review & editing. **Rafia Lodhi:** Writing – review & editing. **Ahmed Eldaidamouni:** Writing – review & editing. **Wesam Hritani:** Writing – review & editing. **Nisreen Haroun:** Writing – review & editing. **Muhammet Hasan:** Writing – review & editing. **Laurence S Sperling:** Writing – review & editing, Supervision, Project administration, Investigation, Funding acquisition, Conceptualization. **Arshed A. Quyyumi:** Writing – review & editing, Supervision, Investigation, Funding acquisition, Conceptualization. **Puja K. Mehta:** Writing – review & editing, Supervision, Methodology, Conceptualization.

## Declaration of competing interest

The authors declare that they have no known competing financial interests or personal relationships that could have appeared to influence the work reported in this paper.
